# Plumbagin-Loaded Glycerosome Gel as Topical Delivery System for Skin Cancer Therapy

**DOI:** 10.3390/polym13060923

**Published:** 2021-03-17

**Authors:** Shadab Md, Nabil A. Alhakamy, Hibah M. Aldawsari, Mohammad Husain, Nazia Khan, Mohamed A. Alfaleh, Hani Z. Asfour, Yassine Riadi, Anwar L. Bilgrami, Md Habban Akhter

**Affiliations:** 1 Department of Pharmaceutics, Faculty of Pharmacy, King Abdulaziz University, Jeddah 21589, Saudi Arabia; nalhakamy@kau.edu.sa (N.A.A.); haldosari@kau.edu.sa (H.M.A.); maalfaleh@kau.edu.sa (M.A.A.); 2 Center of Excellence for Drug Research & Pharmaceutical Industries, King Abdulaziz University, Jeddah 21589, Saudi Arabia; 3 Department of Biotechnology, Jamia Millia Islamia (Central University), New Delhi 110025, India; mhusain2@jmi.ac.in; 4 Department of Pharmaceutics, School of Pharmaceutical Education and Research, Jamia Hamdard, New Delhi 110062, India; khannaziaspn91@gmail.com; 5 Vaccines and Immunotherapy Unit, King Fahd Medical Research Center, King Abdulaziz University, Jeddah 21589, Saudi Arabia; 6 Department of Medical Microbiology and Parasitology, Faculty of Medicine, King Abdulaziz University, Jeddah 21589, Saudi Arabia; hasfour@kau.edu.sa; 7 Department of Pharmaceutical Chemistry, College of Pharmacy, Prince Sattam Bin Abdulaziz University, Al-Kharj 11942, Saudi Arabia; yassinriadi@yahoo.fr; 8 Department of Entomology, Rutgers University, New Brunswick, NJ 08901, USA; anwarbil@scarletmail.rutgers.edu; 9 Faculty of Pharmacy, DIT University, Dehradun 248009, India

**Keywords:** Box–Behnken design, confocal microscopy, liposome, gel, plumbagin, skin cancer

## Abstract

Plumbagin (PLM) is a phytochemical which has shown cytotoxicity against of cancer cells both in vitro and in vivo. However, the clinical application of PLM has been hindered due to poor aqueous solubility and low bioavailability. The aim of the present study was to develop, optimize and evaluate PLM-loaded glycerosome (GM) gel and compare with conventional liposome (CL) for therapeutic efficacy against skin cancer. The GM formulations were optimized by employing design expert software by 3-level 3-factor design. The prepared GMs were characterized in vitro for vesicle size, size distribution, zeta potential, vesicle deformability, drug release, skin permeation, retention, texture, antioxidant and cytotoxicity activities. The optimized formulation showed a vesicle size of 119.20 ± 15.67 nm with a polydispersity index (PDI) of 0.145 ± 0.02, the zeta potential of −27 ± 5.12 mV and entrapment efficiency of 76.42 ± 9.98%. The optimized PLM-loaded GM formulation was transformed into a pre-formed gel which was prepared using Carbopol 934 polymer. The drug diffusion fluxes of CL gel and GM-loaded gel were 23.31 ± 6.0 and 79.43 ± 12.43 µg/cm^2^/h, respectively. The result of texture analysis revealed the adequate hardness, cohesiveness, consistency, and viscosity of the developed GM-loaded gel compared to CL gel. The confocal images showed that glycerosomal gel has deeper skin layer penetration as compared to the control solution. GM-loaded gel treated rat skin showed significantly (*p* < 0.05) higher drug accumulation in the dermis, higher cytotoxicity and higher antioxidant activity as compared to CL gel and PLM suspension. Thus, findings revealed that novel GM-loaded gel could be potential carriers for therapeutic intervention in skin cancer.

## 1. Introduction

Cancer as a life-threatening disease is increasing in prevalence worldwide. It caused an estimated 9.6 million deaths in 2018 with an anticipated mortality of 16.4 million by 2040 [1]. Skin cancer is a complex and common malignancy, spreading worldwide with alarming mortality statistics. The common skin cancers are classified as non-melanoma and melanoma skin cancer. The non-melanoma skin cancer represented by basal cell carcinoma, and squamous cell carcinoma. The skin cancer eruption leads to deformation of skin cells and may result in systemic migration and metastases if not diagnosed promptly [2]. The pathogenesis of skin cancer is multi-factorial, but the prevalent risk factor is long exposure to carcinogenic and inflammatory agent. Indeed, the long exposure to UV radiation (UVA and UVB) also leads to the development of skin cancer due to impairment of genetic material, activating tumor promoter genes, inflammation and oxidative stress [3,4,5]. The current therapeutic approach involves the application of chemotherapy, radiotherapy and surgery.

Skin provides a large surface area for the topical delivery of a vast number of therapeutic formulations. To overcome this barrier, several strategies have been developed. The strategies include polymeric, organic, and inorganic nanoparticles, liposomes, niosomes, carbon tubes, micelles, and quantum dots including, ultrasound-assisted delivery, iontophoresis and electroporation [6,7,8,9]. The drug-loaded nanocarrier passively accumulated neighboring to tumor tissues due to poor lymphatic drainage and vacularization. It provides sustained and local drug delivery to the tumor microenvironment as desirable for treatment of cancer cells. For potential eradication and significant apoptosis of tumor cells, long exposure of therapeutics in specific area of skin is preferably required [4,10]. Conventional liposomes as a vesicular drug delivery system lack the ability to provide sufficient drug release and deeper penetration across the skin, thereby limiting their therapeutic efficacy. This major drawback led to research of novel vesicular carriers resulting in modification of composition of liposomes to increase drug delivery and efficacy [11].

In this context, some authors modified the composition of specially designed vesicular carriers to enhance the permeation and penetration into the deeper layers of skin. Thus, novel, modified vesicular carriers so-called ultradeformable or elastic liposomes such as transferosome, ethosomes, niosomes, and invasomes have been successfully evaluated and introduced in drug delivery applications [12].

Ethosomes, when administered, fuse with the cutaneous layer of skin due to the interaction of ethanol with skin lipid. The permeation of drug is enhanced into the epidermal layer of skin owing to elastic deformation process [13]. However, the alcoholic interaction with lipid molecules of skin leads to decreased transition temperature in stratum corneum. It further leads to phase separation and crystal-phase transformation of solid and liquid lipids. Apart from these, the alcoholic content in ethosome causes skin irritation. On the contrary, GM causes no harm and is completely accepted after topical application [14,15]. Furthermore, >20% glycerol stabilizes the GM vesicle system. The high viscosity and hygroscopic nature improve flexibility and fluidity of the lipid bilayer. This nature allows GM to squeeze through topical barrier resulting in higher drug release and improved permeation and efficacy when used topically or transdermally [16].

Engineered hydrogel mimics the extra cellular matrix of the epidermal tissue of skin due to better structural and mechanical strength. The nanosized hydrogel as polymeric 3D dimensional network in aqueous dispersion is formed by hydrophilic polymer chains that are chemically or physically cross connected [17]. Gels are intelligently explored in active/passive cancer targeting due to their tailor surface, tunable size, ease of preparation and high encapsulation. It is prominently internalized in the target cells, prevents accumulation in the non-target tissues, minimize side effects and reduce therapeutic dose [18].

(5-hydroxy-2-methyl-1, 4-naphthoquinone) extracted from root of plant Plumbago zeylanica having a number of potential therapeutic effects like anti-malarial, anti-microbial [19] and anti-inflammatory effects [10,20]. In a number of cancers, including liver, breast, esophagus, colon, prostate, brain, and lung cancer, plumbagin have shown in vitro/in vivo anti-proliferative and chemo-preventive effects [10,20,21]. The tumor inhibitory mechanism of plumbagin is based on several molecular signaling pathways which are crucial in cell proliferation, invasion, survival and metastasis. It suppresses these major signal transduction pathways STAT3, mTOR/AKT, and NF-κB which plays pivotal role in the growth, development and progress of cancer. Plumbagin retarded UV-ray induced carcinoma in squamous region of skin in mice [22,23,24]. The activation of STAT3 signal pathway is associated 70% case of melanoma cancer [25]. Plumbagin also suppresses hyper-pigmentation of skin by inhibiting an α-melanocyte-stimulating hormone and inhibited tyrosinase activity in B16-F10 melanoma cells [26]. Ti and coworkers also reported that plumbagin do not induce cytotoxicity in normal lens epithelial cells (B3) and normal human keratinocytes (HaCaT) cells at lower concentration (1–5 μM) suggesting plumbagin is safe for skin application [26].

The term “glycerosome” was first acquainted by Manca and associates for topical delivery of diclofenac. It is a versatile drug delivery carrier system which is a modification of liposomes. They are small or large unilamellar or multilamellar lipid vesicles composed of phospholipids, water, and varying concentrations of glycerol (preferably 20 to 30% *w*/*v*). Additionally, they are nontoxic and accepted for topical application [16,27]. The lipid layer in GM is more flexible and possess high fluidity suitable for topical and transdermal drug delivery. The glycerol in these vesicles improves deformability index and thus increases skin permeation and penetration of therapeutics. Cholesterol enhances the stability of GM as well as maintains the lipid membrane integrity by causing barrier to the aqueous phase. When GM dispersed in aqueous phase, phospholipid rapidly arranges themselves as bi-layer vesicles [28,29].

The present study involves the optimization and formulation of PLM-loaded GM using Box–Behnken Design. The independent variables were phospholipid, cholesterol, and glycerol concentration while the dependent variables were vesicle size, entrapment efficiency and drug permeation/flux of PLM, respectively. The optimized formulation was characterized for its size, charge and morphology, texture, drug permeation/flux, drug release, permeation, and retention. Additionally, characterizations based on cytotoxicity were also evaluated. During the study it was speculated that incorporating PLM into GM could enhance drug loading and entrapment with improved aqueous solubility, thereby prolonging systemic availability in sustained-release manner; a combination of all these properties would consequently increase the therapeutic efficacy of PLM and reduce untoward effects.

## 2. Materials and Methods

### 2.1. Materials

PLM, glycerol, rhodamine B and cholesterol were purchased from Sigma-Aldrich (St. Louis, MO, USA). Phospholipid 90 G was procured by Phospholipid GmbH, Germany. The in vitro cell line B16-F10 cells were obtained from National Centre for Cell Science (NCCS) (Pune, India). Dulbecco’s modified Eagle’s medium (DMEM), antibiotics, Foetal Bovine Serum, and MTT reagent were purchased from Gibco (Gaithersburg, MD, USA). Analytical grade was used for HPLC water, solvents and chemicals used in the analysis.

### 2.2. Design and Development of Nanosystem

#### Preparation of PLM-Loaded Nanosystems

The PLM-loaded CLs were developed by thin film hydration technique [27,30]. A weighted amount of PLM (5 mg/mL) was dissolved in chloroform containing ~1% of ethanol along with phospholipid (15.0 mg/mL) and cholesterol (4.0 mg/mL) in a round-bottom flask and mechanically stirred at 40 °C for 1 h. Under reduced pressure, the mixture was evaporated using a rotary evaporator (Buchi Labortechnik AG, New Castle, MA, USA), resulting formation of transparent lipid film around the round bottom flask, and traces of solvent were extracted overnight under vacuum. Further, the lipid film was dried and hydrated at pH 7.4 for 1 h in phosphate buffer saline (PBS) for PLM-loaded CLs. Subsequently, the film suspension was probe sonicated (Hielscher Ultrasonics, Berlin, Germany) for 1.5 min at 90% amplitude and ultracentrifuged at 7500× *g* for 10 min at 4 °C to get rid of unentrapped drug excess in solvent and lyophilized for future application. For PLM-loaded GMs, the obtained lipid films were hydrated with glycerol–water mixture (30% *w*/*v* glycerol) in two successions followed by mechanical agitation for 1 h at 40 °C. Finally, the vesicle dispersions were sonicated for half cycle, i.e., 60 s with 3 s layoff period for every 5 s. The developed formulation centrifuged at 7500× *g* for 10 min at 4 °C to move out unentrapped drug excess, and lyophized for further use.

### 2.3. Box–Behnken Design

Box–Behnken Design Expert (Design-Expert VR Software Version 10, State-Ease Inc., Minneapolis, MN, USA) software was used for optimization of PLM-loaded GMs and CLs [13,31,32]. Based on the design expert software, 17 total runs were generated with 3-level and 3-factor experimental model for optimization of the GMs formulation. The independent and dependent variables are shown in Table 1. Among the various models like 2FI, cubic, linear, and quadratic, the best fit model was analyzed according to ANOVA for statistical design. The significant F-value, low PRESS value, lack of fit (*p* > 0.05) determined the best fitting model.

### 2.4. Characterization of Nanosystem Dispersion

#### 2.4.1. Particle size, Zeta Potential and Morphological Analysis

The Zetasizer-ZSP (Malvern Instruments, Worcestershire, UK) work on the dynamic light scattering was used to evaluate the vesicle size, polydispersity index (PDI) and zeta potential of optimized PLM-loaded CLs, PLM-loaded GMs and rhodamine-loaded glycerosome. The formulations were diluted with deionized water and analysed in triplicate (*n* = 3). The actual size of PLM-loaded GM and CL and were measured by transmission electron microscopy (TEM, JEOL JEM1010, Tokyo, Japan). The drug-loaded GMs and CLs sample (1 mg/mL) was diluted in deionized water. The sample volume of 10 µL was then applied on carbon-coated copper grid. The extra water over copper grid was bumped off and dried. It was then negatively stained with 1% phosphotungstic acid and tested at 10–100-fold enlargements operating at an accelerating voltage of 80 kV under the TEM.

#### 2.4.2. Entrapment Efficiency and Drug Loading

The entrapped amount of PLM in GMs, CLs and fluorescent dye in glycerosome before lyophilization was estimated by high-speed centrifugation system at 7500× *g* for 10 min at 4 °C [33,34]. After centrifugation, the clear aliquot was removed, filtered and PLM content was analyzed by HPLC analysis. In brief, the separation of analyte using HPLC system (Shimadzu, Japan) was achieved on Agilent C_18_ column of dimension 5 µm, 250 mm × 4.5 mm i.d. The other components of HPLC system were quaternary LC-10 AVP pumps, SPD-10AVP HPLC UV-detector. PLM was estimated at a wavelength of 265 nm in binary mobile phase comprised of methanol and sodium dihydrogen phosphate phase (9:1 *v*/*v*) using calibration curve [35]. The calibration curve was constructed in the linearity range of 1–10 µg/mL. The entrapment efficiency of the optimized formulation was calculated by using formula. The obtained numeric for the calibration curve was Y = 0.096 X + 0.021 with regression coefficient value (R^2^ = 0.998).

% Entrapment efficiency = Drug content (A − B)/Total amount of drug × 100

% Drug loading = Drug content (A − B)/Total weight of glycerosome × 100

where A is the total amount of drug and B is the amount of drug analyzed in the super natant using HPLC [34].

#### 2.4.3. In Vitro Drug Release and Kinetic Studies

In vitro drug release study was performed in PBS with an acidic pH 4.5 and PBS pH 7.4. The acidic range is the pH of the skin and pH at the tumor site [36]. The analysis was carried out using dialysis bag techniques. The weighed amount of PLM-loaded GMs, CLs and PLM suspension were placed on the dialysis membrane (MWCO, 12000 Da, Sigma-Aldrich, St. Louis, MO, USA) and immersed in a beaker filled with 100 mL of PBS solution. The beaker placed in a thermostatically controlled shaking water bath at a temperature 37 ± 0.5 °C and release of the drug from formulation was examined at the mentioned pH values separately. At regular intervals of time (0, 1, 2, 4, 6, 8 & 12 h), 3 mL of PBS was withdrawn and was reinstated with the same amount of fresh PBS to maintain sink condition. The samples were analyzed using HPLC. In release kinetic study, drug release profile from formulation was fitted into zero order, first order, Korsmeyer–Peppas, Higuchi, and Hixson-Crowell kinetic models and the best one was discriminated based on the correlation coefficient (R^2^ ~1) value.

### 2.5. Encapsulation of the Nanosystem into a Preformed Gels

The gel base was prepared as the method described in our earlier work [13]. 1.0% *w*/*w* carbopol 934 was continuously stirred for 2 h using magnetic bead in 10 mL of distilled water separately for both PLM-loaded GMs and CLs. Accordingly, propylene glycol, methyl paraben, and triethanolamine were added with uninterrupted stirring until a transparent gel formed. The PLM-loaded GMs and CLs were injected continuously with stirring into the corresponding pre-formed gel base and labeled them as GM-loaded gel and CL gel. The gel texture analyzer was used to measure the texture of developed gels (TA.XT Plus Texture Analyzer, Stable Micro Systems Ltd., Surrey, UK). The spreadability of gels were measured by placing 500 mg of both CLs and GM gels separately in-between the glass slides upto a diameter of 2 cm. Thereafter, the 0.5 kg of weight was placed on the upper glass slide for the duration of 5 min and gels spreading were determined.

### 2.6. Skin Permeation Studies of GM-Loaded Gels

The experiments were performed using fabricated Franz diffusion cell with an effective surface area of diffusion 0.750 cm^2^. The dorsal surface of rat skin was excised with fatty layers removed surgically, washed with alcohol and temporary stored at −80 °C. Before commencing the permeation study, the stored rat skin was equilibrated in PBS for 2 h at room temperature. The Franz diffusion cells decorated the skin specimenssecurely between donor and receptor compartments with the stratum corneum (SC) side facing the donor compartment. Prior to the study, receptor compartment was filled with 7.5 mL of PBS solution and stirred continuously with a small magnetic bead at 500 rpm, maintained at a temperature of 37 ± 0.5 °C. GM-loaded gels, CL gels, PLM suspension (each 1 mL) were placed onto the surface of skin and at regular intervals of time, i.e., 0, 1, 2, 3, 4, 6, 8, 12, 14, 16, 20 and 24 h, 1 mL of solution was withdrawn from the receiving compartment and same amount was replaced with fresh solution. The drug content was analyzed by HPLC analysis.

### 2.7. Drug Retention Study of GM-Loaded Gels

For drug retention study, the mounted skin was removed from Franz diffusion cell, washed, cleaned for adhered drug particles and further subjected to tape stripping technique for removal of SC from dermal layer using scotch crystal tape [37]. Furthermore, the epidermis was separated from dermis by applying surgical sterile scalpel. The tissue protein extracting reagent (T-PER) solution in the ratio of tissue: T-PER (1:10 *w*/*v*) was applied for ameliorating extraction capacity from skin and probe sonicated for 5 min. Henceforth, the tap strips, dermis and epidermis were transferred into methanol, well sonicated to extract the drug and subsequently analyzed by HPLC.

### 2.8. Confocal Microscopy of Rhodamine B-Loaded GM

To validate the drug release and distribution from GMs formulation into different layer of skin, confocal microscopy was performed which required entrapment of rhodamine B fluorescent dye into glycerosome instead of drug. GMs were loaded with 0.02% *w*/*v* fluorescent dye instead of drug in the preparation of GM by thin film hydration technique and applied onto the skin. In this experiment, processed animal skin was mounted on the Franz diffusion cell of which stratum corneum of skin was facing to donor compartment. The rhodamine B-loaded GMs (1 mL) was transferred on the donor compartment and release of probe dye in the receptor was checked for the same duration as performed in skin permeation study. The receptor compartment was filled with 6 mL of PBS, pH 7.4 and the temperature of medium in the diffusion cell was asserted at 32 ± 0.5 °C. Post completion of study, skin was gently wiped with deionized water (HPLC grade) and mounted on the glass slide with a drop of glycerin and observed under confocal microscope with excitation (λex) and emission wavelength (λem) was set at 540 nm and 630 nm applying argon laser beam and 65× objective lens (EC-Plan Neofluar 65×/01.40 Oil DICM27). The distribution and penetration depth of rhodamine B dye into the different layers of skin from GM compared with rhodamine B solution. The z-axis of confocal microscope optically analyzes the fluorescent permeation through the skin layers.

### 2.9. Cytotoxicity Assessment

The cell viability assessment of GM-loaded gels, CL gels, equivalent dose of PLM suspension, blank GMs and blank CLs were performed using MTT assay in murine melanoma cell lines (B16-F10). The mature cells were seeded in 96 well plates (cell density, 5000 cells/well) in 100 µL of Dulbecco’s Modified Eagle’s Medium (DMEM) culture media to allow cell adherence. Subsequently, the cells were incubated in humidified chamber at 37 °C with 100% relative humidity furnished with 5% CO_2_. At the end of 24 h, culture medium was discarded and treated with varying concentrations of 2.5, 5, 7.5, 10 µM of PLM suspension, CL, and GM-loaded gels. Untreated cells were cultured in complete media and considered as control which is 100% cell viable. Accompanied by 24, 48 and 72 h of treatment, 250 µL of MTT reagent was added into well plate and humidified for 2 h. Further, 150 µL of DMSO was added to dissolve formazan crystals (indicating purple colour) as a means of counting viable cells. The absorbance of the specimen on plates was examined on Microplate Reader (BioTek Synergy HT) at 550 nm and % cell viability was determined [38].

### 2.10. In Vitro Radical Scavenging Assay

The DPPH (2, 2-diphenyl-1-picryl-hydrazyl) radical scavenging power of PLM in optimized GM-loaded gels was determined in accordance with the developed protocol [39]. The GM-loaded gels (100 µL) and PLM suspension (100 µL) were mixed with 3.9 mL of 0.025% DPPH solution with vigorous shaking, incubated for 30 min at 28 °C and quantified by UV-spectrophotometry at 517 nm. The free radical scavenging power of optimized GM-loaded gels was estimated corresponding to reduced optical density with control and served as an indication of free radical scavenging power of the formulation. The reduced DPPH concentration was measured by plotting calibration curve of Trolox as standard and the antioxidant power was assessed in TEAC as µg Trolox equivalent/g of sample.

### 2.11. Statistical Analysis

The data analysis was performed using analysis of variance (ANOVA) followed by Tukey Kramer analysis for multiple comparison among the groups using GraphPad Prism 7.00 software. Student’s *t*-test was used as comparison between two groups. The level of significance was considered when *p* < 0.05.

## 3. Results and Discussions

### 3.1. Optimization of PLM-Loaded GM by Statistical Design

Response surface morphology is extensively employed for the optimization of nanoformulation. Among the various statistical experimental designs, Box–Behnken design is an efficient optimization technique used frequently for comparison with conventional optimization procedures. It is largely explored owing to lesser number of experiments and evaluation in optimum time period. The independent variables and their impact on the response used herein for the optimization and development process were based on the significant preliminary observation.

It has been shown that glycerol 10% or lesser produced vesicles with reduced flexibility, prompt deformation and may have lower skin penetration. As the concentration of glycerol increases in the formulation, the increasing elasticity of vesicles were observed and at 30% glycerol concentration the GM were more elastic and resisted deformation. At this concentration glycerol may act as edge activator at phospholipid bilayer [40]. The glycerol content in the GM formulation makes the preparation irritant free on topical application as caused by ethanol in ethosome lipid formulation. The cholesterol in GM formulation is added to ameliorate stability in lipid bilayer, for modifying the surface charge and prevention of vesicle aggregation [16]. The experimental runs with % compositions of individual components and their responses are recorded in Table 2.

The dependent variables were subjected to numerical models fit analysis, for their individual, interaction and quadratic effects. The different models, i.e., cubic, linear, 2FI and quadratic models, the best fitted model was found to be quadratic model. The optimum value of different responses vesicle size, entrapment efficiency, and flux were recorded on the criteria of desirability. The regression analysis of responses, vesicle size (Y_1_), entrapment efficiency (Y_2_) and flux (Y_3_) for fitting to quadratic model are expressed in Table 3.

**Figure 1:**
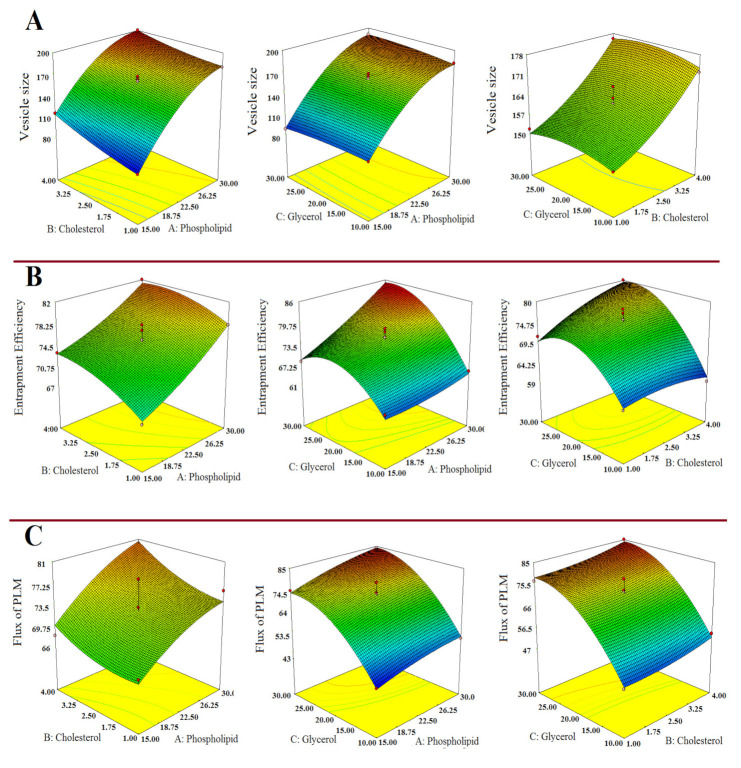
Response surface morphological plots indicating the comparative effects of independent variables, i.e., Phospholipid conc. (mg); Cholesterol conc. (mg); and Glycerol conc. (% *w*/*v*) on (**A**) Vesicles size, (**B**) % Entrapment efficiency and (**C**) Flux of PLM.

**Figure 2:**
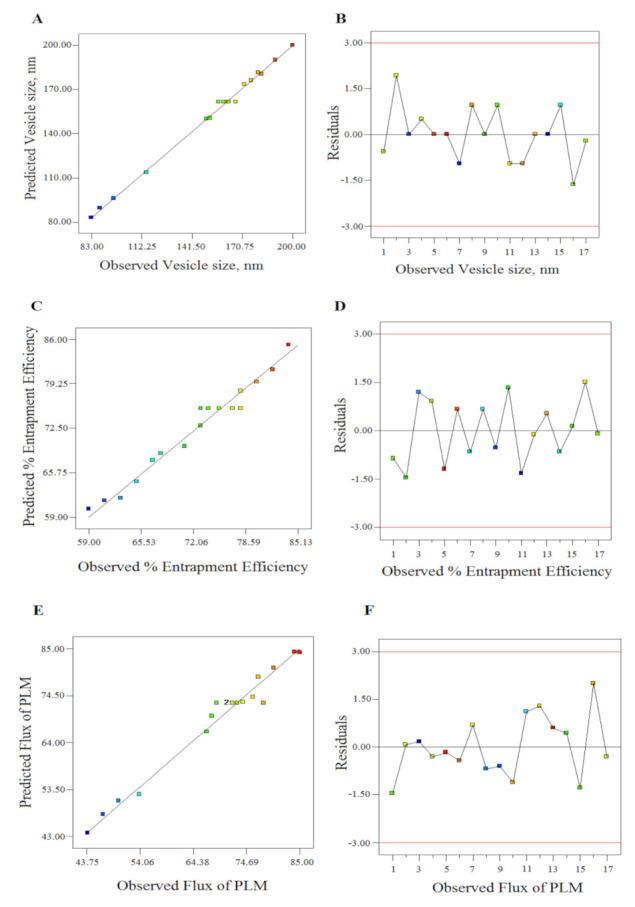
The linear correlation plots (**A**,**C**,**E**) between predicted vs. observed values and corresponding residual plots (**B**,**D**,**F**) for responses vesicle size, entrapment efficiency, and flux of plumbagin (PLM).

The 3-D response surface curve showing relative effects of independent variables on responses Y_1_, Y_2_ and Y_3_ are established in Figure 1A–C. The statistical plot expressing the correlation between actual vs. predicted value for the responses and their residual plot are indicated in Figure 2A–F.

#### 3.1.1. Impact of Independent Variables on Vesicle Size (Y_1_)

The impact of phospholipid concentration, cholesterol and glycerol concentration on vesicle size could be explicated by quadratic polynomial equation as shown in Table 3. It was worth noting that the Model F-value of 360.55 implicated that the model was significant. The “Lack of Fit F-value” of 0.94 implies it was insignificant. The “Pred R_2_” of 0.99 is in reasonable agreement with the “Adj R2” of 0.99 as shown in Table 3.

The phospholipid concentration had significant positive impact on vesicles size of GMs *(p* < 0.05). The vesicles size analysis of developed formulation revealed the size ranges from 82.23 ± 4.78 nm to 200 ± 18.40 nm. At low concentrations, the observed vesicle sizes were 82.23 ± 4.78 nm and upon increasing the phospholid concentration to 22.5 mg, the maximum increase in vesicle size was 176 ± 16.80 nm (FL13). Further increase in phospholipid concentration to 30 mg led to maximum increase in vesicle size to 200.00 ± 18.40 nm (FL6), as shown in Table 2. The result was observed similar of prior reported method [13].

The cholesterol concentration had positive impact on vesicles size of GMs. The increasing cholesterol concentration from 1.5 to 4.0% *w*/*v* led to an increase in vesicle size. Although, at same concentration of cholesterol, substantial decrease in vesicle size was observed as in FL14 formulation which could be due to combined effect of phospholipid and glycerol concentrations Table 2.

The glycerol concentration had less positive impact on the vesicle size. The formulation FL8 had vesicle size of 182 ± 14.40 nm at 10% *w*/*v*. Likewise, formulation FL9 and FL11 of same glycerol concentration (10% *w*/*v*) had vesicle size of 150 ± 12.70 and 172 ± 16.80 nm, respectively. In spite of the aforementioned result, small vesicle size of 96 ± 9.40 nm was observed in formulation FL3 at 10% *w*/*v* of glycerol concentration which could be due to combined impact of phospholipid and cholesterol concentrations. At 20% *w*/*v* of glycerol concentration FL1, FL2, FL4 and FL17 had vesicle size of 160 ± 16.00 nm, 167 ± 17.10 nm, 163 ± 15.20 nm and 161 ± 13.50 nm, respectively. Similar result was observed in formulation FL12 of vesicle size 180.3 ± 15.40 nm. Further, a substantial decrease in vesicle size was observed in FL14 at same concentration of glycerol which might be due to combining effect of phospholipid and cholesterol concentrations. The increasing aqueous glycerol concentration led to increased vesicle probably due to sticky texture of glycerol [27].

#### 3.1.2. Impact of Independent Variables on % Entrapment Efficiency (Y_2_)

The derived quadratic equation for % entrapment efficiency (%EE) as shown in Table 3. The Model F value of 26.93 implies that the model is significant. The “Lack of Fit F-value” of 0.64 implies it was insignificant. The “Pred R_2_” of 0.82 was in reasonable agreement with the “Adj R2” of 0.93. The entrapment efficiency of optimized PLM-loaded GM formulation ranges in between 59 ± 4.20% to 84 ±9.10%. The increased phospholipid concentration led to increased %EE of PLM-loaded GM due to signifcant positive impact (*p* < 0.05) on it as indicated in Table 2. The cholesterol concentration had positive impact on the %EE. It was observed that increase in concentration of cholesterol from 1 to 2.5% *w*/*v* resulted enhanced entrapment of formulations as seen in Table 2. Moreover, at higher cholesterol concentration (4% *w*/*v*) mixed effect was observed for formulations FL6, FL11, FL13 and FL15. The formulation FL6 and FL13 showed improved %EE of 82 ± 8.60% and 80 ± 7.80%, while FL15 and FL11 depicted decreased %EE, i.e., 73 ± 6.80% and 59 ± 4.20%, respectively. This is probably due to combined impact of different concentrations phospholipid, glycerol and cholesterol. The glycerol concentration had significant positive impact on EE (*p* < 0.05). It was observed that increasing glycerol concentration from 10% to 30% *w*/*v* increased the %EE from 59 ± 4.20% to 84 ± 9.10%. However, in some formulations, FL7 and FL10 at 30% *w*/*v* concentration of glycerol the %EE siginficantly dropped to 68 ± 7.40% and 71 ± 7.40% due to negative quadratic effect of glycerol (*p* < 0.05). All the obervations were reported in agreement with the previous studies [41].

#### 3.1.3. Impact of Independent Variables on Flux (Y_3_)

The derived quadratic equation for flux is as shown in Table 3. The “Model F-value” of 32.59 suggested that the model is significant. The “Lack of Fit F-value” of 0.69 implies that the “Lack of Fit” is insignificant. The “Pred R2” of 0.86 was in reasonable agreement with the “Adj R2” of 0.94. The phospholipid concentration had siginficant positive impact on flux of PLM. It was ascertained that the flux of PLM steps up on increasing the phospholipid concentration from 15 to 30 mg in the formulations. The PLM-loaded formulation with phsopholipid concentration range from 15 to 30 mg had shown flux of 74 ± 5.80 μg/cm^2^/h to 84 ± 9.90 μg/cm^2^/h, respectively.

The cholesterol concentration has low positive impact on the flux. At low cholesterol concentration 1.0% *w*/*v* the formulation FL10 expressed flux as 77.0 ± 8.60 μg/cm^2^/h whereas further increasing cholesterol to 2.5% *w*/*v* the maximum increase in flux was observed in formulation FL5 as 84.0 ± 9.90 μg/cm^2^/h. It was examined that increasing the glycerol concentration leads to an increase in the flux due to significant positive impact of PLM flux (*p* < 0.05). At initial concentration of glycerol (10% *w*/*v*) the flux of PLM for formulation FL3 was 44.0 ± 4.30 μg/cm^2^/h and further increasing the glycerol concentration from 20 to 30% *w*/*v*, the flux increases from 67.0 ± 6.70 μg/cm^2^/h in formulation (FL14) to 85.0 ± 9.90 μg/cm^2^/h (FL13).

The optimized formuation of GMs comprised of phospholipid concentration, (15 mg), cholesterol concentration (4.00 mg) and glycerol (26.8% *w*/*v*). The design expert software revealed the predicted values of dependent variables, i.e., vesicle size, entrapment efficiency and flux of PLM was 110.53 nm, 74.09% and 76.12 μg/cm^2^/h, respectively. Further, the selected optimized formulation experimentally performed and evaluated for vesicle size, entrapment efficiency and flux were found to be 119.20 ± 15.67 nm, 76.42 ± 9.98% and 79.43 ± 12.43 μg/cm^2^/h. The percentage error reported between observed vs. predicted values of vesicle size, entrapment efficiency and flux were 8.18, 3.14, and 4.34 within the acceptable range (Table 4). It was noticed that the experimental values were established closer to the predicted values established by design expert software. The vesicle appeared marginally bigger than predicted vesicle size by design expert reasonably due to lyophilized state of formulation. In addition, the PDI and zeta potential value of optimized formulation was found to be 0.145 ± 0.02, zeta potential −27 ± 5.12 mV. The low value of PDI substantiated narrow size distribution, consistent and homogeneous nature of formulation [42]. The surface negative charge shown by zeta potential studies over optimized formulation clarified it exits over the surface of vesicle which may be contributed to by cholesterol or glycerol used in the formulation. The negative charge over vesicle surface further assured the de-aggregation of vesicles due to electrostatic repulsion and that resistive force may facilitate in enhancing the bio-stability of nanovesicles [43].

### 3.2. Characterization of Nanosystems Dispersion

#### 3.2.1. Particle Size, Zeta Potential, Morphology, Entrapment and Drug Loading Efficiency

**Figure 3:**
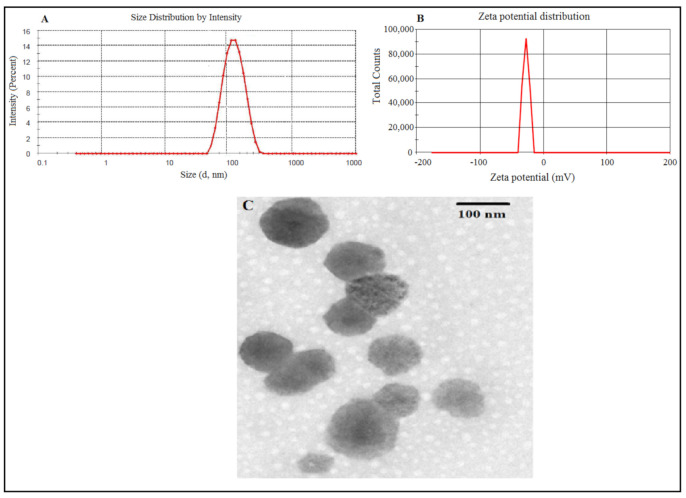
Optimized PLM-loaded glycerosomes (**A**) average vesicle size (**B**) zeta potential and (**C**) transmission electron micrograph.

A mean vesicle size of 119.20 ± 15.67 nm with uniform size distribution and PDI value of 0.145 ± 0.02 (Figure 3A) was optimized for the developed glycerosomal formulation. The vesicle surface was negative since the zeta potential value was analysed to be −27 ± 5.12 mV as shown in Figure 3B. The TEM image of GMs indicated that the particles were uniform in size, consistent, spherical and homogenously dispersed as depicted in Figure 3C. The observed entrapment efficiency and drug loading for optimized PLM-loaded GMs were 76.42 ± 9.98 and 7.64 ± 1.12%, respectively. The in vitro characterization of PLM-loaded CLs in terms of size, PDI, surface charge, entrapment, drug loading efficiency and transmission electron micrograph are provided in (Supplementary Sheet Table S1 & Figure S1).

#### 3.2.2. In Vitro Drug Release and Kinetic Study

**Figure 4:**
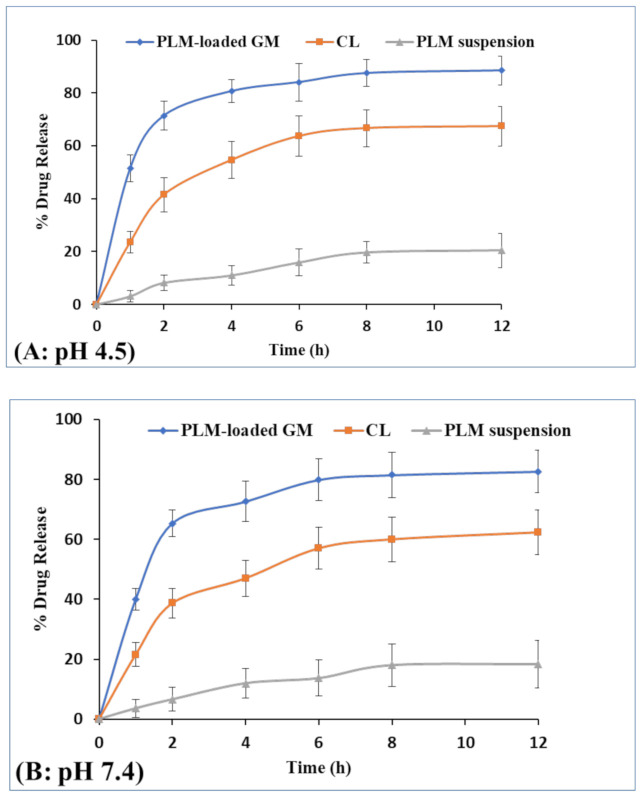
Percent drug release of PLM from PLM-loaded glycerosomes, CLs and PLM suspension in PBS of pH 4.5 (**A**) and 7.4 (**B**), respectively.

The release profiles of PLM-loaded GMs, CLs and PLM suspension in PBS (pH 4.5, 7.4) is expressed in Figure 4A,B. The release study was conducted for 12 h and it was observed that PLM release pattern from GM was biphasic, i.e., initial burst release accompanied with sustained release pattern. In the first hour, release of PLM from PLM-loaded GMs, CLs and PLM suspension at pH 4.5 were found to be 51.5 ± 5.0%, 23.6 ± 4.0% and 3.1 ± 1.0%, respectively. On the other hand, the PLM release from PLM-loaded GM, CL and PLM suspension at pH 7.4 for first hour were found to be 40 ±3.6%, 21.5 ± 4.0% and 3.6 ± 0.4%, respectively. The result has shown significant differences (*p* < 0.05) in the drug release profile of PLM-loaded GM at pH 4.5 and 7.4 for first hour. The PLM released from PLM-loaded GMs, CLs and PLM suspension at 12 h were observed to be 88.5 ± 5.5%, 67.4 ± 7.5% and 20.4 ± 6.5%, respectively. Further, drug release at pH 7.4 from PLM-loaded GM, CL and PLM suspension were 82.6 ± 7.0%, 62.3 ± 7.5% and 18.3 ± 8.0%. The result has shown small increase in drug release at acidic pH as compared to pH 7.4. However, the result has shown no significant differences (*p* > 0.05) in the drug release profile of all formulations at pH 4.5 and 7.4 over 12 h. The release of PLM from GM and CL was more at acidic pH 4.5 than at pH 7.4 probably due to enhanced solubility of aggregated drugs inside vesicles. However, the significant differences (*p* < 0.05) were observed in percent release of PLM from GM as compared to CL and PLM suspension over 12 h at pH 4.5 and 7.4. The obtained dissolution profile of PLM was comparable to earlier cited work based on the transferrin conjugated PLM liposome [44]. The drug release profile was fitted into various release kinetic models (Zero order, first order, Higuchi, and Hixon Crowell) to screen out the best fitted model as shown in Table S2. Based on R2 value, the best fitted model for PLM-loaded GMs was Higuchi model with R2 value of 0.9758 [32]. The release mechanism of PLM-loaded GMs was analyzed by applying Korsmeyer-Pappas model and the value of n exponent was found 0.69, i.e., in between 0.5 to 0.89 which represent that PLM released follow non-fickian diffusion mechanism [45].

#### 3.2.3. Characterization of Gels

The prepared GM-loaded gels were good in consistency, appearance, as well as texture. The pH value of developed formulation was tested and was found to be 7.40 ± 0.03 which was within the acceptable range, hence free from any form of irritancy [33]. The texture analysis of prepared gel was subjected to force curve plot. The results obtained after analysis of GM-loaded gels formulation expressed as firmness or hardness value, viscosity index, consistency and cohesiveness were found to be 212.70 g, 221 g.s, 63.54 g.s and −151.60 g, respectively. On the other hand, for CL gel firmness or hardness value, viscosity index, consistency and cohesiveness were found to be 219.30 g, 230.32 g.s, 39.08 g.s and −160.72 g, respectively. The spreadability of CL gels and GM-loaded gels were measured and 1.34 ± 0.16 cm and 2.74 ± 0.28 cm, respectively. Therefore, texture testing of the developed GM-loaded gel is substantially accommodated for topical application because of its good consistency, cohesiveness, firmness, and viscosity compared to CL gel [13].

#### 3.2.4. Skin Permeation Study

**Figure 5:**
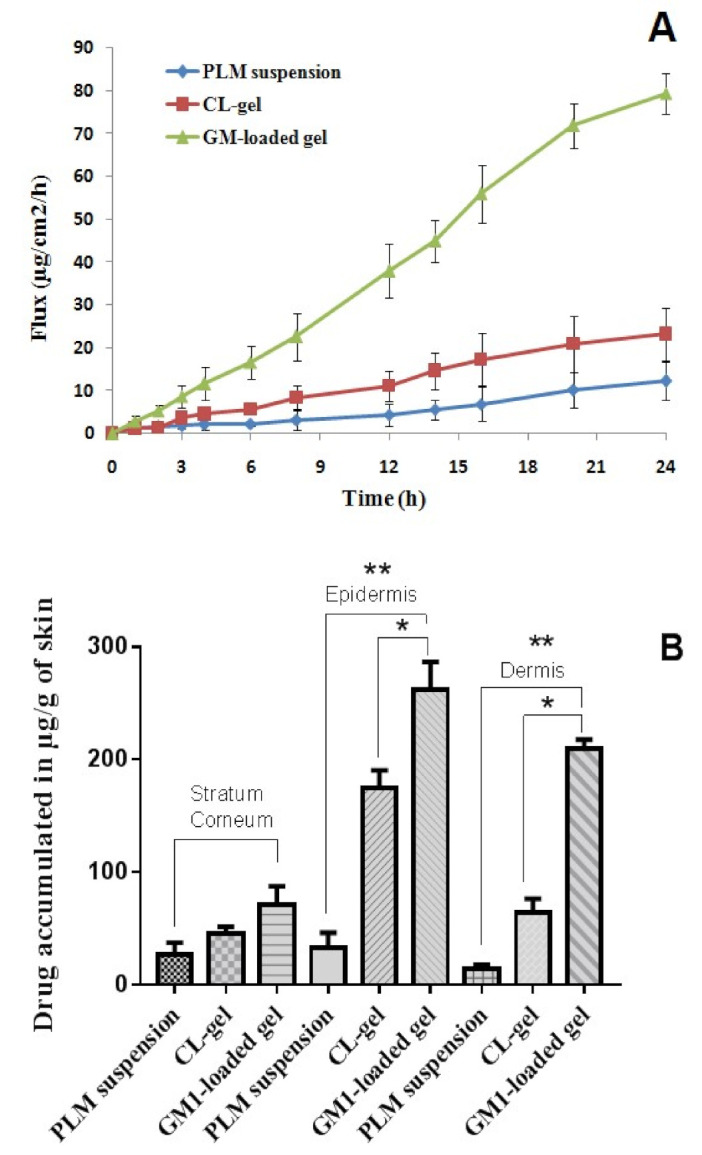
(**A**) Represents skin permeation studies in which flux of PLM suspension, CL gel and GM-loaded gel were compared and (**B**) The amount of drug retained in stratum corneum, epidermis and dermis from free PLM, CL gel, and GM-loaded gel, respectively. Data expressed as mean ± SD (*n =* 3) (* *p* ≤ 0.05, ** *p* ≤ 0.01).

The skin permeation and penetration ability of drug from GM-loaded gel was carried out using animal skin as shown in Figure 5A. The flux was determined over a period of 24 h and calculated from slope of linear part of the graph. The maximum flux at end point of the study from GM-loaded gels, CL gels and PLM suspension were 79.43 ± 12.43, 23.31 ± 6.0, and 12.3 ± 4.5 µg/cm^2^/h. The outcome of the study ascertained that flux of PLM from GM-loaded gels was statistically significant when compared with flux of PLM from CL gel (*p =* 0.0159) and PLM suspension (*p* = 0.0006). The high degree of permeation from the glycerosomal gel clearly indicated the presence of glycerol in the vehicle which makes the vesicle ultra-deformable and elastic in nature thereby allowing the drug to penetrate deeper to cutaneous layer and dermal tissues. Poor drug solubility, permeation as well as cutaneous barrier through the strata of skin are some of the possible reasons for obtaining low flux from PLM suspension.

#### 3.2.5. Drug Retention Study

The amount of drug retained in the stratum corneum, epidermis, and dermis were analyzed by HPLC and expressed in µg/g of skin tissues. The amount of PLM from suspension, PLM from CL gels and GM-loaded gels deposited in the SC were 37, 50.8 and 89 µg/g of skin tissue. Further, the amount of PLM from suspension, CL gels and GM-loaded gels deposited in the epidermis were 45, 188.4 and 289 µg/g of skin. Moreover, the dermis had 17, 73.98 and 210 µg/g of skin tissue of PLM from suspension, CL gels and GM-loaded gels as shown **in** Figure 5B. In comparison to drug suspension and CL gels, significant amount of PLM was deposited in the epidermis and dermis from GM-loaded gels (*p* < 0.05) which was probably due to edge activating effect and ultra-deformable and elastic behavior of GM containing glycerol.

#### 3.2.6. Confocal Laser Microscopy

**Figure 6:**
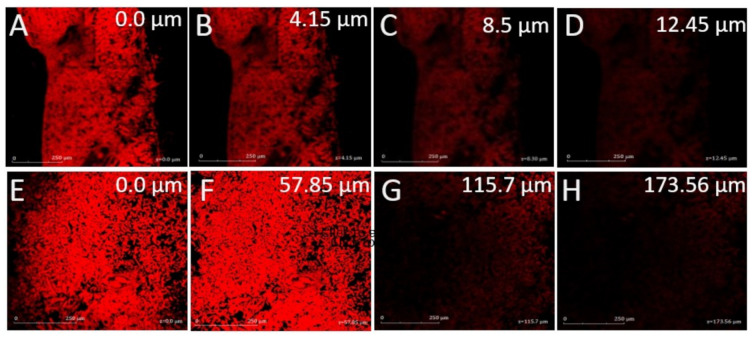
Confocal laser microscopy of (**A**–**D**) Rhodamine B solution and (**E**–**H**) Rhodamine B-loaded glycerosome, scale bar = 250 µm.

The depth of penetration from both Rhodamine B solution and Rhodamine B encapsulated GMs were compared. The microscopy revealed that the maximum depth of penetration from Rhodamine B solution was 12.45 µm while Rhodamine B encapsulated GM formulation reached upto 173.56 µm deeper in the soft tissues of skin as indicated in Figure 6. The high degree of penetration from Rhodamine B encapsulated GM was probably owing to fusion of vesicle content with different layers of skin, followed by disruption of vesicle and enhanced fluidity of lipidic layer that led to eventual distribution of Rhodamine B. In fact, the glycerol was able to modify the fluidity of lipid bilayer in the vesicle and easily squeezed through microscopic pores into the deeper soft layers of skin [46].

#### 3.2.7. MTT Assay

**Figure 7:**
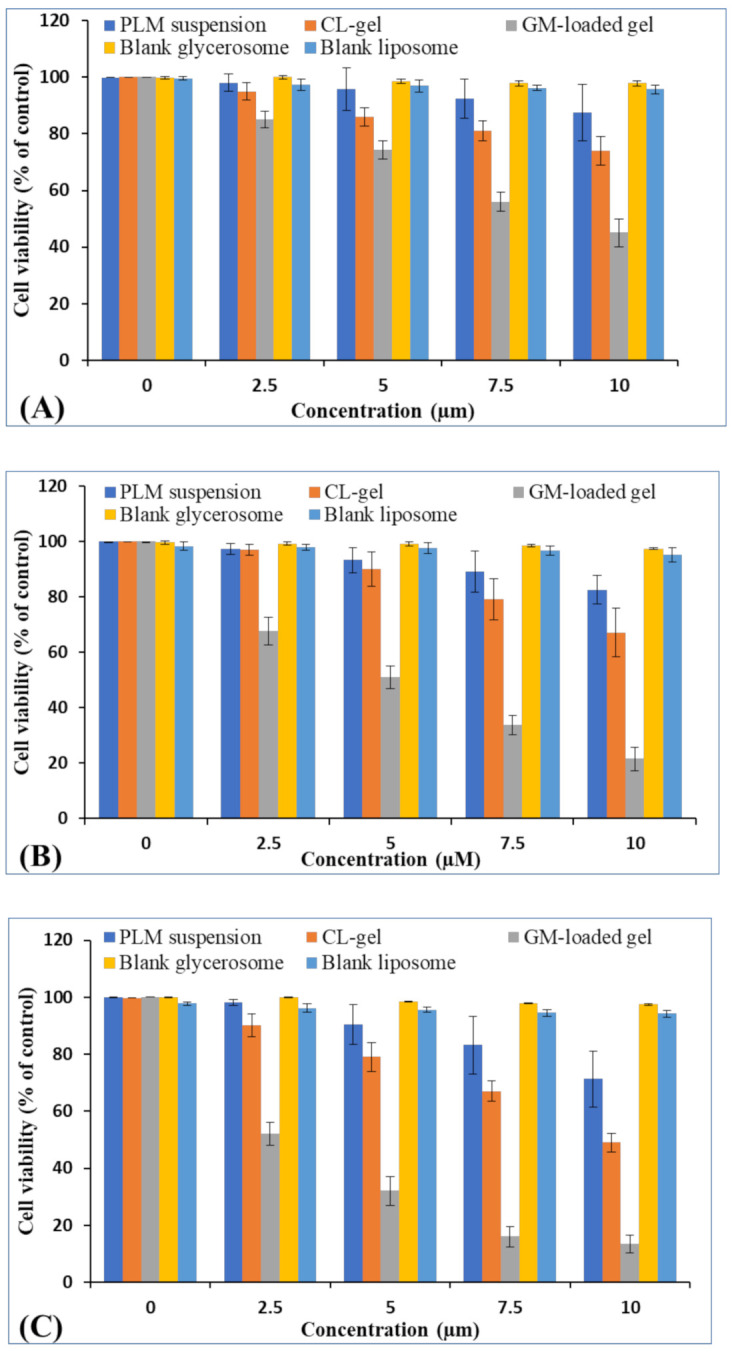
The cell viability of PLM suspension, CL and GM-loaded gel, blank GM and blank liposome post 24 h (**A**) 48 h (**B**) and 72 h (**C**) in cancer cell line. Data expressed as mean ± SD (*n =* 3).

The cytotoxic assessment was performed via MTT assay on murine tumor cell line (B16-F10) for human skin cancer. As per the assay, the cell viability (%) after treatment with PLM suspension, CL gels and GM-loaded gels was obtained. The outcomes of the MTT assay demonstrated that GM-loaded gels showed significantly enhanced cytotoxicity when compared to PLM suspension and CL gels Figure 7A–C. It was observed that cytotoxic assessment was concentration and time dependent because as the concentration and time of exposure increased, better apoptosis of cells took place. Blank formulation of GMs and CLs did not show any sign of cytotoxicity. The IC_50_ values of PLM suspension, CLs and GM-loaded gels are as shown in Table 5. The outcomes evidently show that GM-loaded gel had significantly higher cytotoxic effect than CL and PLM suspension (*p* < 0.05) over different periods of incubation. The high concentration of PLM was passively transported to inside the cells thus leading to cytotoxic effect. Further, the nanovehicle releases drug intracellularly through various transporters in the extracellular matrix present on the cell membrane [47].

#### 3.2.8. Antioxidant Activity

**Figure 8:**
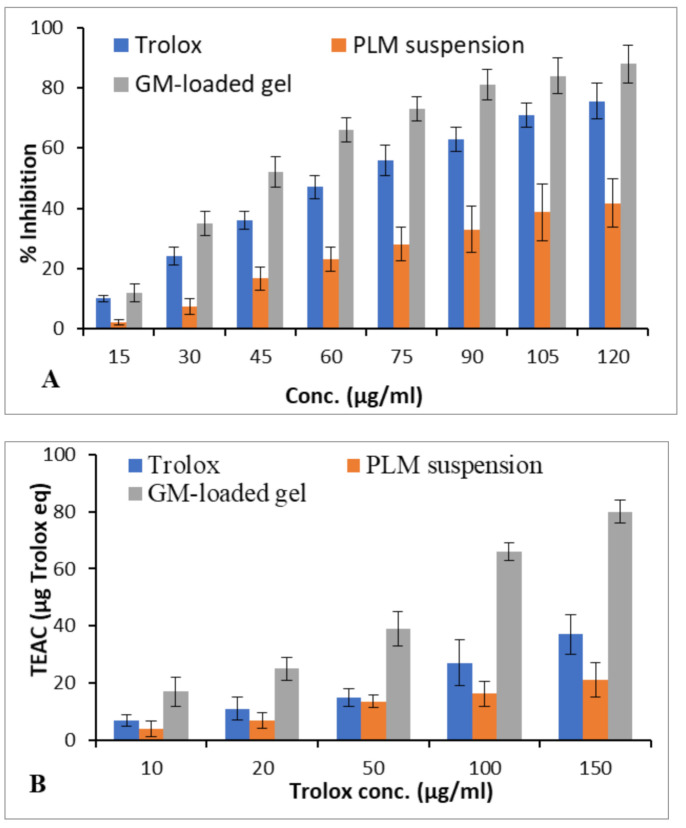
The antioxidant activities govern by DPPH assay of PLM suspension and GM-loaded gel. Percentage inhibitory effect of antioxidant (**A**). Trolex equivalent antioxidant activity (TEAC) of GM-loaded gel compared with trolox and PLM suspension (**B**). Values shown as means ± SD (*n =* 3) analysed by one way ANOVA followed by Bartlett’s test for statistical significance (*p* < 0.05).

The antioxidant activity was evaluated using DPPH assays. The estimation of Trolox equivalent antioxidant capacity (TEAC) was performed from the standard curve of Trolox. The TEAC value of optimized GM-loaded gels was 17, 25, 39, 66, and 80 µg trolox equivalent per 10, 20, 50, 100, and 150 µg/mL of trolox in DPPH assay (Figure 8). The GM-loaded gels showed significantly higher antioxidant activity than PLM suspension (*p* < 0.05) [48].

## 4. Conclusions

The novel formulation of GM-loaded gels was successfully developed and compared with CLs formulation and PLM suspension. The GM formulation was optimized using Box–Behnken statistical design and successfully studied the effects of independent variableson dependent variables. The optimized formulation was expressed as nano-size vesicle, negatively charged ZP and high entrapment efficiency. The developed formulation exhibited sustained release and gave excellent flux across various strata of cutaneous layer. The confocal laser microscopy revealed considerably good and deep penetration of GM inside the cutaneous layers. Cytotoxic assessment of GM-loaded gels showed significant cytotoxicity over CL and PLM suspension. The antioxidant study of PLM from GM-loaded gels demonstrated pronounced antioxidant effect as compared to CL and PLM suspension. Overall, the study proved that PLM containing GM-loaded gels could be a promising carrier in skin cancer therapy.

## Figures and Tables

**Table 1:** Box–Behnken design experimental dependent and independent variables with levels (low, medium and high) employed in the fabrication of PLM-loaded GMs.

Independent Variables	Level Used		
	Low (−1)	Medium (0)	High (+1)
X_1_: Phospholipid conc. (mg)	15	22.5	30
X_2_: Cholesterol (mg)	1	2.5	4
X_3_: Glycerol	10	20	30
**Dependent variables**			
Y_1_: Vesicles size (nm)	Minimize		
Y_2_: Entrapment efficiency (%)	Maximize		
Y_3_: Drug permeation/Flux (μg/cm^2^/h)	Maximize		

**Table 2:** Formulations with composition of individual independent variables and observe responses of PLM-loaded glycerosome in Box–Behnken design.

Formulation No	Independent Variables			Observe Responses		
	X_1_ (mg)	X_2_ (% *w*/*v*)	X_3_ (% *w*/*v*)	Y_1_ (nm)	Y_2_ (%)	Y_3_
FL1	22.50	2.50	20.00	160.00 ± 16.30	74.00 ± 3.20	69.00 ± 6.80
FL2	22.50	2.50	20.00	167.00 ± 17.10	73.00 ± 5.30	73.00 ± 6.10
FL3	15.00	2.50	10.00	96.00 ± 9.40	63.00 ± 7.50	44.00 ± 4.30
FL4	22.50	2.50	20.00	163.00 ± 15.20	77.00 ± 8.60	72.00 ± 6.50
FL5	30.00	2.50	30.00	190.00 ± 18.30	84.00 ± 9.10	84.00 ± 9.90
FL6	30.00	4.00	20.00	200.00 ± 18.40	82.00 ± 8.60	80.00 ± 8.60
FL7	15.00	2.50	30.00	88.00 ± 6.60	68.00 ± 7.40	74.00 ± 5.80
FL8	30.00	2.50	10.00	182.00 ± 14.40	65.00 ± 6.60	50.00 ± 4.60
FL9	22.50	1.00	10.00	150.00 ± 12.70	61.00 ± 6.90	47.00 ± 4.60
FL10	22.50	1.00	30.00	152.00 ± 10.50	71.00 ± 7.40	77.00 ± 8.60
FL11	22.50	4.00	10.00	172.00 ± 16.80	59.00 ± 4.20	54.00 ± 5.90
FL12	30.00	1.00	20.00	180.00 ± 15.00	78.00 ± 9.50	76.00 ± 9.50
FL13	22.50	4.00	30.00	176.00 ± 16.80	80.00 ± 7.80	85.00 ± 9.90
FL14	15.00	1.00	20.00	82.23 ± 4.70	67.00 ± 7.60	67.00 ± 6.70
FL15	15.00	4.00	20.00	115.00 ± 8.50	73.00 ± 6.80	68.00 ± 7.90
FL16	22.50	2.50	20.00	157.00 ± 10.80	78.00 ± 7.30	78.00 ± 8.80
FL17	22.50	2.50	20.00	161.00 ± 13.50	75.30 ± 9.20	72.00 ± 8.30

X_1_: Phospholipid (mg); X_2_: Cholesterol (%*w*/*v*); X_3_: Glycerol (% *w*/*v*); Y_1_: Vesicles size (nm); Y_2_: Entrapment efficiency (%); Y_3_: Drug diffusion Flux (μg/cm^2^/h).

**Table 3:** Regression analysis summary for various responses Y_1,_ Y_2_, and Y_3._

Response Surface Quadratic Model	R-Squared	Adj R-Squared	Pred R-Squared	Adeq Precision	PRESS	% CV	Mean	SD
Response 1 (y_1_)	0.9966	0.9923	0.9851	48.695	302.25	2.05	152.47	3.13
Response 2 (y_2_)	0.9719	0.9358	0.8294	17.237	150.61	2.60	72.25	1.88
Response 3 (y_3_)	0.9767	0.9467	0.8689	18.105	334.87	4.24	68.82	2.92
$\mathrm{Vesicle}\;\mathrm{size}\;\left(Y_{1}\right)=161.60+46.25\times X_{1}+12.25\times X_{2}+0.75\times X_{3}-3.00\times X_{1}\times X_{2}+4.00\times X_{1}\times X_{3}+0.50_{}\times X_{2}\times X_{3}-20.30\times X_{1}{}^{2}+3.20\times X_{2}{}^{2}-2.30\times X_{3}{}^{2}$								
$\mathrm{EE}\;\left(Y_{2}\right)=75.46+4.75\times X_{1}+2.13\times X_{2}+6.88\times X_{3}-0.50\times X_{1}\times X_{2}+3.50\times X_{1}\times X_{3}+2.75\times X_{2}\times X_{3}+0.89\times X_{1}{}^{2}-1.36\times X_{2}{}^{2}-6.35\times X_{3}{}^{2}$								
$\mathrm{Flux}=72.80+4.63\times X_{1}+2.50\times X_{2}+15.63\times X_{3}+0.75\times X_{1}\times X_{2}+1.00\times X_{1}\times X_{3}+0.25\times X_{2}\times X_{3}-1.40\times X_{1}{}^{2}+1.35\times X_{2}{}^{2}-8.40\times X_{3}{}^{2}$								

**Table 4:** Composition, experimental vs. predicted value with percentage error of optimized PLM-loaded glycerosome formulation.

Variables	Optimum Composition	Response	Observed Value of Response	Predicted Value of Response	Percentage Error
X_1_	15 mg	Y_1_	119.20 ± 15.67	110.53	8.18
X_2_	4 mg	Y_2_	76.42 ± 9.98	74.09	3.14
X_3_	26.8% *w*/*v*	Y_3_	79.43 ± 12.43	76.12	4.34

Predicted error (%) = (observed value − predicted value)/predicted value × 100%; X_1_, Phospholipid (mg); X_2_, Cholesterol *(% v*/*v*); X_3_, Glycerol, Y_1,_ Vesicles size (nm); Y_2_, Entrapment efficiency; Y_3_, Drug permeation/Flux (μg/cm^2^/h).

**Table 5:** IC_50_ value of PLM suspension, CL gel and GM-loaded gel containing PLM in B16-F10 cell line.

Incubation Time (h)	PLM Suspension (µm)	CL Gel (µm)	GM-Loaded Gel (µm)
24	41.7 ± 2.3	19.0 ± 1.2	8.9 ± 0.6
48	29.8 ± 1.4	16.0 ± 1.3	5.6 ± 0.4
72	19.1 ± 1.7	10.4 ± 0.8	4.1 ± 0.3

## Supplementary Materials

The following are available online at https://www.mdpi.com/2073-4360/13/6/923/s1, Table S1. The particle size, PDI, zeta potential, entrapment efficiency and drug loading of PLM-loaded glycerosome, PLM-loaded CL and rhodamine-loaded glycerosome; Figure S1. Diagram showing (A) Average size of vesicle 129.56 ± 17.05 nm; PI 0.245 ± 0.032, (B) zeta potential −22.50 ± 5.61 (mV), and (C) Transmission electron micrograph of optimized PLM-loaded liposome formulation; Table S2. Drug release kinetic analysis of optimized PLM-loaded GM formulation.
