# Supplementary material for: Plumbagin-Loaded Glycerosome Gel as Topical Delivery System for Skin Cancer Therapy
Source: Polymers (Basel). 2021 Mar 17;13(6):923. doi: 10.3390/polym13060923 (PMC8002493; doi:10.3390/polym13060923)
Supplement: Supplementary file 1 [file polymers-13-00923-s001.pdf]

Supplementary data sheet

Table 1S. The particle size, PDI, zeta potential, entrapment efficiency and drug loading of PLM-loaded glycerosome, PLM-loaded CL and rhodamine-loaded glycerosome

Characterizations	PLM loaded CL	PLM-loaded glycerosome	Rhodamine-loaded glycerosome
Vesicle size (nm)	129.56 ± 17.05	119.20 ± 15.67	121.50 ± 13.67
PDI	0.245 ± 0.032	0.145 ± 0.02	0.155 ± 0.03
Zeta potential (mV)	-22.50 ± 5.61	-27 ± 5.12	-25 ± 6.33
Entrapment efficiency (%)	71.67 ± 11.08	76.42 ± 9.98	75.31 ± 9.78
Drug loading (%)	3.06 ± 0.12	7.64 ± 1.12	-

1. Characterization of PLM-loaded conventional liposomes

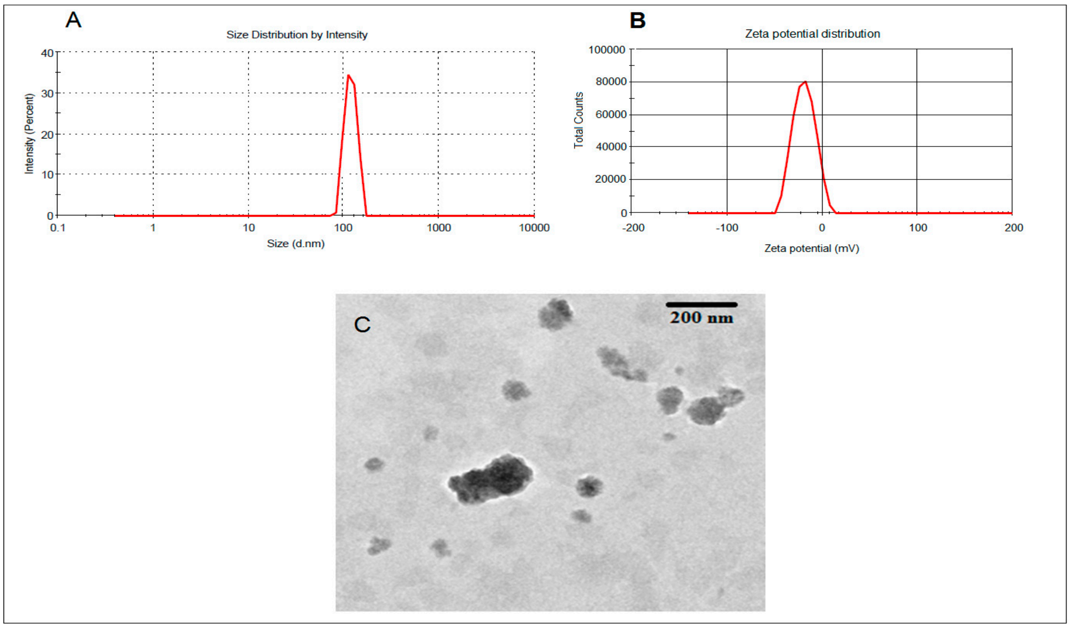

Figure S1. Diagram showing (**A**) Average size of vesicle 129.56 ± 17.05 nm; PI 0.245 ± 0.032, (B) zeta potential -22.50 ± 5.61 (mV), and (**C**) Transmission electron micrograph of optimised PLM loaded liposome formulation.

Table S2. Drug release kinetic analysis of optimized PLM-loaded GM formulation

Model	n	k	R^2
Zero order	1.00	0.316	0.9027
First order	0.698	0.667	0.7240
Higuchi	0.492	0.018	0.9758
Korsmeyer-Pappas	0.670	1.679	0.9495
Hixson Crowell	0.732	1.278	0.8421
